# Antimicrobial and anticarcinogenic activity of bioactive peptides derived from abalone viscera (*Haliotis fulgens* and *Haliotis corrugata*)

**DOI:** 10.1038/s41598-023-41491-w

**Published:** 2023-09-13

**Authors:** Crisalejandra Rivera-Pérez, Xolotl Paloma Ponce González, Norma Yolanda Hernández-Savedra

**Affiliations:** Centro de Investigaciones Biológicas del Noroeste, S.C., Instituto Politécnico Nacional 195, C.P. 23096 La Paz, BCS México

**Keywords:** Biochemistry, Biotechnology

## Abstract

Bioactive peptides have been studied in several sources due to their valuable potential in the pharmaceutical and food industries. Abalone viscera, which are normally discarded as byproducts, are a rich source of protein. Thus, the aim of this study was to explore the potential bioactivity of peptides derived from abalone viscera (*Haliotis fulgens* and *Haliotis corrugata*) after hydrolysis with a commercial mixture of enzymes. The hydrolysates obtained were fractionated using gel filtration chromatography. The resulting hydrolysate fractions were investigated for their antimicrobial and cytotoxic activities, including the expression of gelatinases *mmp-2* and *mmp-9* in human prostate cancer cell lines (PC3). Results showed antimicrobial activity for protein fractions of *H. corrugata* against *Proteus mirabilis* and *Pseudomona aeuroginosa* (66.2–116.25 kDa), *Bacillus subtilis* (6.5–21.5 kDa), and *Aspergillus niger* (97.4–116.25 kDa), while *H. fulgens* peptide fractions (200–31 kDa) displayed activity against six bacterial strains, and fractions from 116.25 to 21.5 kDa had effects on the fungus *A. niger*, *Alternaria alternata*, and *Aspergillus flavus*. Additionally, protein fractions displayed cytotoxic activity, inhibiting 30.4–53.8% of PC3 cellular growth. Selected fractions decreased the PMA-induced and not-induced expressions of *mmp-2* and *mmp-9* in PC3 cells. Abalone viscera, as byproducts, can be used as a potential source of antimicrobial and anticancer peptides.

## Introduction

Marine organisms represent an important source of bioactive compounds due to their development in a wide range of environments, which requires adaptation mechanisms. These mechanisms include the synthesis of bioactive molecules that function as signaling molecules, defense against predators, elimination of competitors, and inhibition of bacterial and/or fungal invasion^1^. Additionally, these compounds are synthesized in high concentrations to allow for their excretion into the aqueous medium and to ensure their efficacy. Currently, over 3,000 compounds (e.g., proteins, polysaccharides, pigments, etc.) with novel bioactivity have been described from marine sources^2^.

The main sources of marine bioactive compounds are bacteria, cyanobacteria, algae, sponges, cnidaria, bryozoans, mollusks, tunicates, echinoderms, and marine sneaks^1^. However, only a few of them have been characterized and commercialized. Examples of these include ziconotide and brentuximab vedotin, which are marine peptides derived from mollusks *Conus magus* and *Dolabella Auricularia*, respectively. These peptides were approved by the Food and Drug Administration (FDA) in 2011^3–5^. Trabectedin, isolated from a sea squirt (*Ecteinascidia turbinate*), was approved for use against sarcoma by the European Union (EU) in 2007 and by the FDA in 2015^6^. E*ribulin mesylate,* derived from a marine sponge (*Halichondria okadai*), received FDA approval for use against breast cancer in 2010 and EU approval in 2011^7^.

Additionally, a wide variety of marine compounds have been used for diagnostic and experimental purposes. For instance, green fluorescent protein (GFP) isolated from the jellyfish *Aequorea victoria* is used for molecular labeling^8^. Limulus amebocyte lysate (LAL), derived from the crab *Limulus polyphemus*, is utilized for detecting lipopolysaccharides from gram-negative bacteria^9^. Hemocyanin KLH, obtained from the marine mollusk *Megathura crenulata*, serves as a vaccine carrier for treating bladder carcinoma^10^. Furthermore, compounds like tetrodotoxin, okadaic acid, and palitoxin are toxins used in the study of ion channels in eukaryotic cells. Additionally, there are 20 other candidates in phase I, I/II, II or III clinical trials^1^. Therefore, marine organisms offer promising prospects for the discovery of new bioactive molecules that could be valuable in the nutraceutical industry, pharmacology, and research.

The abalone (*Haliotis* spp.) fishery is highly valued as a seafood delicacy in many parts of the world, especially in Asia. Thus, the global production of farmed abalone has significantly increased in recent years^11^. However, during the abalone processing, the viscera, which account for 15–25% of their wet weight, are often discarded^12^. This results in the annual disposal of tons (t) of viscera. For instance, in Mexico alone, approximately 42.9–71.5 t of viscera are generated from an annual fishery production of 286 t^13^. Therefore, there is potential for the valorization of abalone viscera byproducts, as they are a rich source of protein and can be used as precursors of bioactive peptides^14^. There is substantial evidence of bioactive compounds in abalone, such as peptides and polysaccharides, that exhibit various bioactivities, including nutraceutical properties^15^ and pharmacological properties^16–27^.

Given the valuable properties of bioactive peptides, it is hypothesized that peptides derived from hydrolyzed abalone viscera could exhibit antimicrobial activity against various pathogens and cytotoxic effects against cancer cells. The hypothesis is based on previous research that has demonstrated the presence and bioactivity of bioactive compounds, such as peptides, in mollusk viscera. For example, mollusk visceral extracts have demonstrated effectiveness against *Escherichia coli* K1, *Pseudomona aeruginosa*, *Acinetobacter baumanii* (*Perna viridis*^28^, as well as *Vibrio harvey* (*Anadara granosa*^29^). Furthermore, the administration of abalone viscera to mice with breast cancer has shown significant inhibition in tumor progression by suppressing epidermal growth factor (EGF), vascular endothelial growth factor (VEGF), cyclooxygenase-2 (Cox-2), and fibroblast growth factor (FGF)^23^. Some of these factors are regulated by matrix metalloproteinases (MMPs, also known as gelatinases), which are zinc-dependent endopeptidases involved in a variety of pathological processes such as tumor invasion and metastasis^30^. Gelatinases (MMP-2, MMP-9) play an important role in cancer invasion and metastasis and have been detected in human fibrosarcoma cell line HT1080 and PC-3 cells^31,32^. A high level of *mmp-2* and *mmp-9* gene expression has been linked to an unfavorable prognosis in human cancer cell lines, including prostatic adenocarcinoma^33^. Thus, *mmp-2* and *mmp-9* gene expression works as indicators of a favorable prognosis in patients with some types of cancer.

The increase in bacterial resistance, resulting from the widespread use of antimicrobials for disease control, and the rising cases of cancer diseases have created and urgent need for the discovery of novel antimicrobial and anticancer molecules to combat drug resistance and cancer.

Therefore, the aim of this study is to investigate the antimicrobial activity of visceral extracts from the two commercial produced abalone species in Mexico (*H. fulgens* and *H. corrugata*) against 11 opportunistic pathogens from the bacterial family Enterobacteriacea, which are responsible for a wide range of infections. Additionally, four common fungal species are included in the evaluation. Moreover, the anti-cancer activity of visceral extracts was evaluated using a grade IV prostatic adenocarcinoma PC-3 (ATCC CRL-1435™) cell line, which represent the second most commonly occurring cancer in men.

## Materials and methods

### Organisms

Abalone viscera from blue (*Haliotis fulgens*) and green abalone (*Haliotis corrugata*) were donated by the Sociedad Cooperativa Punta Abreojos, S.C. of C.V. in northern Baja California Sur, Mexico.

### Cell lines

The cell line, grade IV prostatic adenocarcinoma PC-3, was obtained from the American Type Culture Collection (ATCC CRL-1435) by the Centro de Investigacion y Asistencia en Tecnologia y Diseño del Estado de Jalisco (CIATEJ).

### Protein extract and enzymatic digestion

Three batches of viscera, weighing 150 g each, were used from blue and green abalone, respectively. The protein from the viscera was extracted following the method described by Nguyen^31^ with some modifications. In brief, digestive organs were homogenized using a blender, and the pH was adjusted to 5.6^34^ using a 150 mM citric acid-sodium citrate buffer, reaching a final volume of 500 mL. An aliquot, 1 mL of each extract was collected for further analysis. Enzymatic hydrolysis of the crude extract was performed using Wobenzym (Societe des Produits Nestle S.A. Vevey Switzerland) (Supplementary Table I). Prior to this, enzyme pills were pulverized and added to the crude extract. Enzymatic digestions were carried out using 0.4, 0.6, 0.8, 1, 10, 12.5, and 15 g of pulverized Wobenzym pill. The mixture was then incubated at 37 °C in a water bath for 4 and 24 h, respectively, with constant agitation. Subsequently, the enzymatic hydrolysates were centrifuged at 10,000 *g*, − 4 °C for 20 min (Beckman, Mod. Js-HS). The soluble fraction was collected and further centrifuged at 100,000 *g*, − 4 °C for 60 min (Beckman, Mod. L7 Ultracentrifuge). The resulting soluble fraction was collected and filtered using sterile Whatman 541 paper (VWR) to remove lipids and non-hydrolyzed molecules. The hydrolyzed extracts were evaluated using SDS-PAGE electrophoresis.

### Protein quantification

Protein quantification of the visceral and hydrolyzed extracts, as well as the chromatographic fractions, was performed using the methods described by Bradford^35^ and Lowry^36^. This was done using a spectrophotometer SmartSpec 3000 (Bio-Rad, Hercules, CA, USA), and a microplate reader (Bio-Rad Benchmark). Bovine serum albumin was used as standard.

### Sodium dodecyl sulfate–polyacrylamide gel electrophoresis (SDS-PAGE)

Protein separation by SDS-PAGE was performed following the method described by Laemmli^37^ using a Mini Protean II system (Bio-Rad, 2000, CA, USA). Samples were mixed with 4 × sample buffer containing 50 mM Tris–HCl pH 6.8, 20% glycerol, 10% SDS, 10% β-mercaptoethanol and 0.05% bromophenol blue. The samples were then boiled for 10 min and loaded into either 10% or 15% polyacrylamide gel. A broad-range molecular weight standard (Bio-Rad, 1610317, CA, USA) was also loaded into the gel. Electrophoresis was carried out at 90-V and room temperature using a vertical electrophoresis unit (Bio-Rad Protean II, CA, USA). After electrophoresis, the gels were stained with Coomassie blue solution (0.05% (w/v) Coomassie Brilliant Blue R-250, 7% v/v acetic acid, 40% v/v methanol) for 2 h. Protein bands were then visualized by soaking the gels in a distaining solution (7% v/v acetic acid and 40% v/v methanol) for 2 h. Gel images were digitalized using a gel imager (Chemi Doc XRS, Bio-Rad, CA, USA). The relative molecular mass of protein bands was determined by comparing their migration with the molecular weight standard.

### Protein fractionation by gel filtration chromatography

Proteins obtained from enzymatic hydrolysis, 2 mL each from green and blue abalone, were subjected to high-resolution gel filtration chromatography using a Sephacryl S-100 column (Cytiva Lifesciences, USA) with dimensions of 1.0 × 31 cm. Prior to sample loading, the column was pre-equilibrated with 150 mM citric-citrate sodium buffer (pH 5.6). Protein fractions were eluted from the column using the same buffer at a flow rate of 0.8 mL/min. A total of fifty-three fractions, each with a volume of 2.4 mL, were collected. Protein peak fractions were determined based on their absorbance at 280 nm. All samples were preserved at − 4 °C until further use.

### Antimicrobial activity

Antimicrobial activities of the visceral extracts and chromatographic protein fractions were assessed using a radial diffusion method in agar, following the protocols described by Fothergill^38^ and Valgas^39^. The bacteria and the filamentous fungi used in the experiments are listed in Table 1. Petri dishes containing tryptic soy agar (TSA) and potato dextrose broth (PD) medium were inoculated with 100 µL of bacteria (1 × 10^8^ cell/mL) and fungi (0.4–5.0 × 10^4^ spore/mL), respectively. Afterward, paper filter disks (Whatman, 1/4, VWR) were placed in the Petri dishes, and 20 µL of hydrolyzed visceral extracts or chromatographic protein fractions were added to their respective filter disks. The agar plates were then incubated at 37 °C, and the growth of bacteria was monitored after 72 h, while fungal growth was monitored after 48 h. The presence of antimicrobial activity was determined by measuring the diameter of the zone of inhibition that appeared around the filter disk following the incubation period. Positive controls for bacteria included gentamicin (0.1 mg/mL), kanamycin (0.1 mg/mL), penicillin (20 U/mL), and streptomycin/penicillin (20 U/mL). For fungi, positive controls consisted of amphotericin (0.4, 4.0, 25.0, 33.0, 50.0, and 100 mg/mL), ketoconazole (0.4, 4.0, 25.0, 33.0, 50.0, and 100 mg/mL), and fluconazole (3 and 15 mg/mL). As a negative control, citric-citrate sodium buffer (150 mM, pH 5.6) was used.

**Table 1:** Bacterial and fungal strains used for antimicrobial activity.

Species	ATCC
Bacteria	
*Proteus mirabilis*	12953
*Shigella sonnei*	9290
*Shigella flexneri*	12022
*Pseudomona aeruginosa*	27853
*Salmonella thyphimurium*	14028
*Salmonella thyphi*	
*Enterobacter aerogenes*	
*Salmonella enteritidis*	
*Escherichia coli*	12228
*Staphylococcus aureus*	25923
*Bacilus subtilis*	6633
Fungi	
*Mucor sp*	LPM
*Alternaria alternata*	LPMAa
*Aspergillus niger*	LPMAn
*Aspergillus flavus*	LPMAf

*LPM* Laboratorio de Patogénesis Microbiana, La Paz Baja California Sur.

### Cytotoxic activity

The chromatographic fractions, 1.5 mL each, were lyophilized. Prostate cancer cells PC-3 (CRL-1435) were cultured in F12K medium (Gibco Life Technologies, Eggenstein, Germany), supplemented with 10% fetal bovine serum (FBS, Gibco), and antibiotics (1% penicillin/streptomycin, Gibco Life Technologies, Eggenstein, Germany)^31,40^. The cells were maintained in a humid atmosphere with 5% CO_2_ at 37 °C and passaged serially when the cell density reached 8 × 10^6^ cells per box. For instance, PC-3 cells become confluent after 4 days, and the number of cells obtained from one T-75 flask was 8 × 10^6^ cells. On the 4th day, the cells were trypsinized from the flask and plated in wells for the assay.

### MTT assay

PC3 cells (1 × 10^4^ cells/well) were seeded into 96-well plates. Briefly, PC3 cells were detached from the box using 0.25% trypsin (Gibco Life Technologies, Eggenstein, Germany) by a 5 min incubation at 37 °C. Then, trypsin was inactivated by adding 5 mL of fresh medium. The cells suspension was recovered after centrifugation at 1500 rpm for 10 min. The supernatant was discarded, and the pellet (cells) was re-suspended in 1 mL of medium. The number of cells was estimated using a Neubauer chamber. Then, the PC3 cells were seeded into a 96-well plate and incubated for 24 h at 37 °C to allow cell adhesion to the plate. Afterwards, the medium from each well was discarded, and 100 µL of protein fractions were added. The protein concentration used was according to Nguyen^31^. Two hundred and 400 µg/mL were added to each well, respectively, followed by a 24 h incubation at 37 °C. Subsequently, 20 µL MTT reagent (5 mg/mL) was added into each well, and the plate was further incubated for 24 h at 37 °C. Finally, 100 µL of DMSO was added to the medium to dissolve the formazan crystals, and the mixture was incubated for 15 min at 37 °C. The formazan was then measured at a wavelength of 595 nm. The plates were read on a microplate reader (Bio-Rad, Bechnmark) before the data were analyzed. The survival rate was calculated as follows: survival rate (%) = 100 × (OD 595 value of tested sample)/OD 595 value of untreated cells).

### MMP-2 and MMP-9 expression

The expression of gelatinases *mmp-2* and *mmp-9* was measured according to Van-Ta^40^ and Nguyen^31^. Cultured PC3 cells were seeded into 48-well plates at a density of 2 × 10^5^ cells/well. The cells were cultured in F12K without FBS for 24 h at 37 °C and 5% humidity. Afterwards, the medium was removed, and chromatographic fractions (300 µg/mL) from *H. fulgens* were added and incubated for one hour, followed by incubation with PMA (10 ng/mL) for 36 h. After incubation, the culture medium was removed, and cells were lysed on the plate using RLT (RNeasy Mini Kit, QIAGEN, Cat No. 74104) for RNA extraction. Samples were collected and stored at − 80 °C for further total RNA extraction.

### RNA extraction and droplet digital PCR (ddPCR)

Total RNA was isolated from the PC3 cells using RNeasy Mini Kit (QIAGEN, Cat No. 74104), following the manufacturer´s instructions. The concentration and purity of the RNA were determined by spectrophotometry using a NanoDrop ND-2000, measuring absorbance at 260/280 and 260/230 nm absorbance. The RNA integrity was assessed by running it on a 1% (w/v) agarose/synergel agarose gel. To ensure the absence of DNA contamination, a direct PCR was performed using 1 µL (50 ng/µL) of each RNA preparation with 28S ribosomal specific primer, a non-amplified control. Subsequently, 8 ng of total RNA was used from each verified RNA sample for cDNA synthesis using the ImProm-II Reverse Transcription System (Promega, Madison WI) and oligo-dT primer, following the manufacturer´s instructions.

The expression of *mmp-2* and *mmp-9* was determined using the droplet digital PCR System (QX200 Droplet Digital PCR System fromBio-Rad). Briefly, each 20 µL reactions consisted of 1X EvaGreen ddPCR Supermix (QX200 ddPCR EvaGreen Supermix, Cat. No. 1864033, Bio-Rad), 100 nM gene-specific primers (Table 2), and 1 µL of the cDNA sample (100 ng). The reaction mixture was combined with 70 µL of Droplet Generation Oil (Bio-Rad), partitioned into 14,000–17,000 droplets in QX200 Droplet Generator (Bio-Rad), transferred to 96-well plates, and sealed. PCR amplification was carried out in a C1000 Touch Thermal Cycler (Bio-Rad) under the following cycling conditions: 1 cycle at 95 °C for 5 min, 40 cycles at 95 °C for 30 s, 60 °C for 30 s, 72 °C for 30 s, 1 cycle at 4 °C for 5 min, 90 °C for 5 min with 2 °C/s ramp rate. Immediately after the end-point amplification, the fluorescence intensity of individual droplets was measured using the QX200 Droplet Reader (Bio-Rad). Data analysis was performed using the QuantaSoft droplet reader software (Bio-Rad). The absolute transcript level was calculated in copies/µL PCR.

**Table 2:** Primers used for *mmp-2* and *mmp-9* gene expression analysis.

Primer ID	Primer sequence (5′ → 3′)	Product size (bp)
*mmp2*-Fw	5´-ACATCAAGGGCATTCAGGAG-3´	133
*mmp2*-Rv	5´-GATCTGAGCGATGCCATCAA-3´	
*mmp9*-Fw	5´-TTTGACAGCGACAAGAAGTGG-3´	133
*mmp9*-Rv	5´-CGGCACTGAGGAATGATCTAA-3´	

### Statistical analysis

Statistical analyses were performed using the GraphPad Prism Software (San Diego, CA, USA). The results are expressed as average ± s.d. Significant differences (p < 0.01) were determined with a one tailed students t-test performed in a pair wise manner.

## Results

### Optimization of hydrolysis parameters for abalone viscera

**Figure 1:**
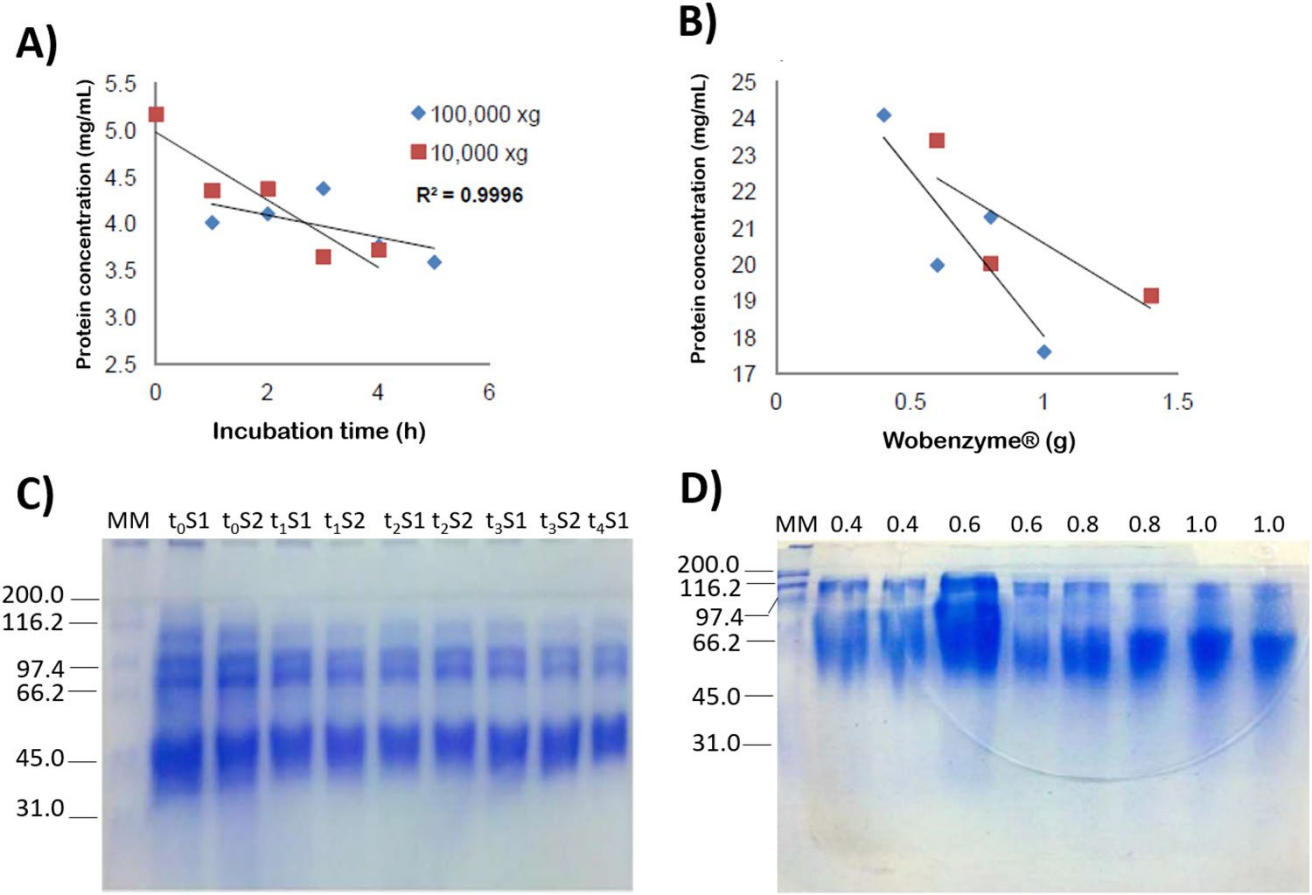
Optimization of protein recovery from *Haliotis fulgens* viscera after hydrolysis with Wobenzym (Societe des Produits Nestle S.A. Vevey Switzerland). (**A**) and (**C**) Effect of incubation time on protein recovery after hydrolysis using 2 g of Wobenzym and two centrifugation conditions. (**B**) and (**D**) Protein recovery after hydrolysis with different amounts of Wobenzym (g). Protein recovered after 10,000 × *g* (represented by red square or S1) and 100,000 × *g* (represented by blue diamond or S2). 10% sodium dodecyl-sulfate polyacrylamide gel electrophoresis (SDS-PAGE). MM: Molecular marker. t_0_ − _4_: Time of hydrolysis of viscera from abalones. Amount of Wobenzym for hydrolysis (0.4–1.0 g). The image represent the full gel of samples runned, more information is presented in the Supplementary Material.

Protein viscera from abalones (*H. fulgens* and *H. corrugata*) were hydrolyzed using Wobenzym (Societe des Produits Nestle S.A. Vevey Switzerland). Optimization parameters for protein extraction were performed using viscera from *H. fulgens* due to the limited number of samples collected for *H. corrugata.* This is because the abalone fishery, mainly supported by *Haliotis fulgens* and *H. corrugata,* is currently experiencing a decline. The optimization process involved two different centrifugation conditions: 10,000 × *g* at − 4 °C for 20 min and 100,000 × *g* at − 4 °C for 60 min. Furthermore, the incubation time of hydrolysis (2, 3, and 4 h) and enzyme concentration (ranging from 0.4 to 1 g/mL) after 4 h of hydrolysis were evaluated (Fig. 1).

No difference was observed in protein recovery when using two different centrifugation conditions (Fig. 1A). Thus, a centrifugation condition of 10,000 × *g* was used for all the subsequent protein recovery extractions. Hydrolysis of the viscera using 2 g of Wobenzym (Societe des Produits Nestle S.A. Vevey Switzerland) for 4 h incubation resulted in a reduction in protein concentration and increased protein hydrolysis, as indicated by SDS-PAGE analysis (Fig. 1C). Increasing the amount of Wobenzym used for visceral hydrolysis led to a higher degree of molecular protein hydrolysis; however, protein bands remained within a wide range of 45–200 kDa (Fig. 1D). To enhance visceral protein hydrolysis, Wobenzyme amounts of 10, 12.5, and 15 g were used for hydrolysis at 37 °C for 24 h. Elevated protein hydrolysis was observed with 10 and 15 g of Wobenzym (Table 3). Nonetheless, no differences were observed in the protein pattern band patterns on SDS-PAGE (Supplementary Fig. 1).

**Table 3:** *Haliotis corrugata* and *H. fulgens* protein concentration after hydrolysis with Wobenzym after 24 h incubation.

Species	Wobenzym^®^ (g)	Protein (mg/mL)
*H. corrugata*	10	14.0
*H. fulgens*	10	15.5
	15	18.0

Wobenzym (Societe des Produits Nestle S.A. Vevey Switzerland).

### Protein fractionation by gel filtration

**Figure 2:**
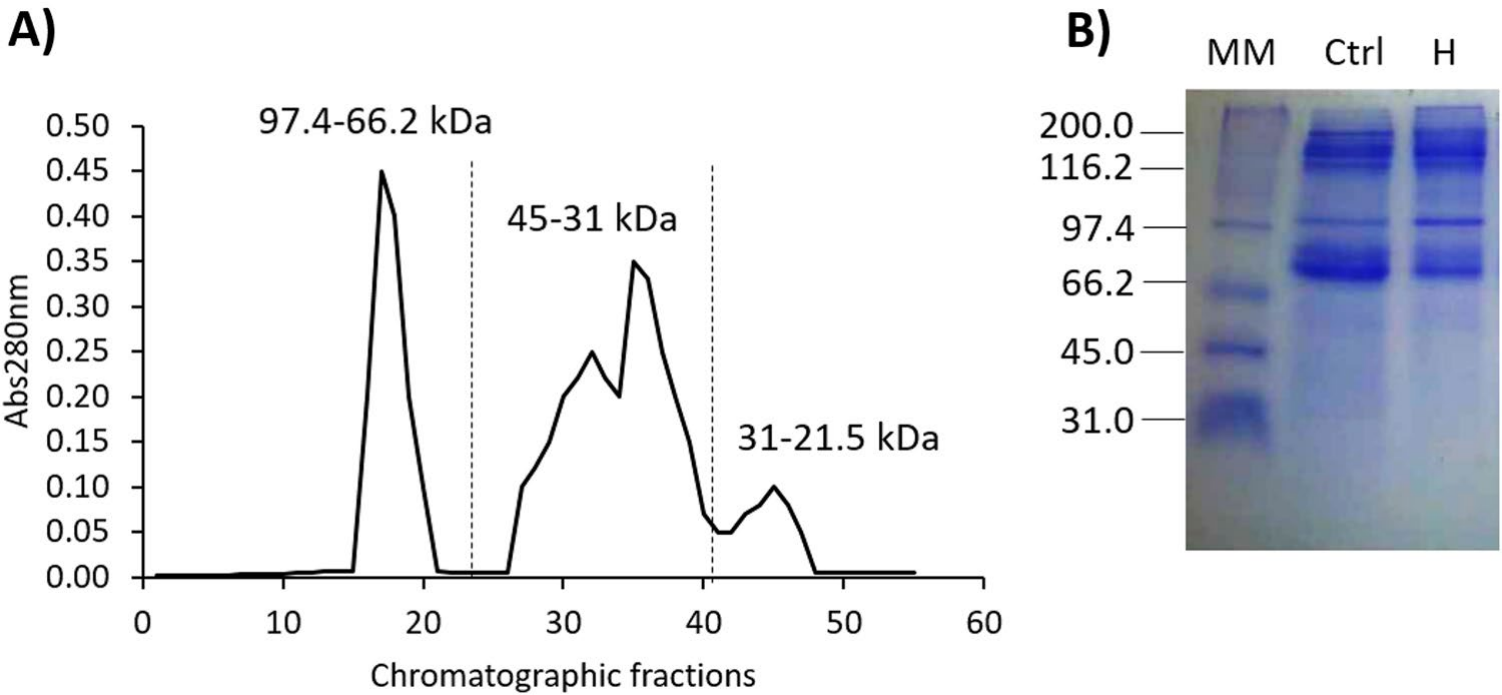
Gel filtration chromatographic profile of hydrolyzed *Haliotis* sp. abalone samples. (**A**) Representative chromatographic profile of visceral fractionation from abalone by gel filtration chromatography using Sephacryl S-100 at a flow rate of 0.8 mL/min and sample volume of 2 mL. Protein was eluted with 150 mM citric-citrate sodium buffer at pH 5.6. (**B**) 15% SDS-PAGE of unhydrolyzed (Ctrl) and hydrolyzed viscera from abalones (H). Chromatographic and SDS-PAGE are representative of three independent experiments. Gel was cropped for concise presentation; the full-length gels is presented in the Supplementary Material.

The hydrolyzed samples with Wobenzym (Societe des Produits Nestle S.A. Vevey Switzerland) shown in Table 1 were used for protein fractionation through gel filtration. Each hydrolyzed viscera, containing 36 mg of total protein, underwent fractionation. A total of 53 fractions were collected. Three protein peaks were observed at the absorption of 280 nm, representing high molecular weight (116.25–66.2 kDa), medium molecular weight (45–21.5 kDa), and low molecular weight (21.5–6.5 kDa) proteins (Fig. 2). The fractions corresponding to each peak were pooled and used for antimicrobial and cytotoxic activity assays.

### Antimicrobial and antifungical activity

**Figure 3:**
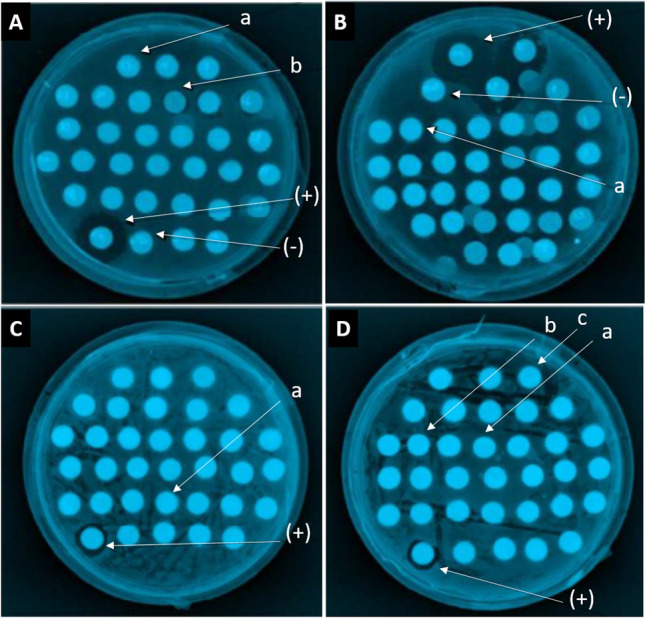
Antimicrobial activity of visceral fractions from *Haliotis fulgens* by agar diffusion assay of four selected bacterial strains. (**A**) *Bacillus subtilis*, a: 200–97 kDa, b: 97.4–66.2 kDa; (**B**) *Escherichia coli*, a: 200–11,625 kDa; (**C**) *Pseudomona aeuroginosa*, a: 45–31 kDa; (**D**) *Staphylococcus aureus*, a: 60.0 kDa, b: 66 kDa, c: 45–31 kDa. Positive controls: gentamycin (0.1 mg/mL), negative controls: 150 mM citric citrate buffer pH 5.6.

Fractions obtained from the hydrolyzed viscera of abalones were tested against nine Gram-negative and two Gram-positive bacteria, as outlined in Table 4 and Supplementary Table II. The concentration used for antimicrobial and antifungical activity for *H. corrugata* was 12 mg/mL due to the lack of viscera sample obtained for the peptide generation. The results showed that the abalone fractions exhibited activity against five Gram-negative and two Gram-positive bacteria (Fig. 3). However, no antimicrobial activity was observed against *S. sonnei*, *S. flexneri*, *S. thyphimurium,* and *S. enteritidis*. Fractions derived from *H. corrugata* containing proteins ranging from ~ 66.2 to 116.25 kDa displayed antimicrobial activity against *P. mirabilis* and *P. aeruginosa*, while low molecular weight proteins (6.2–21.5 kDa) demonstrated sensitivity only against *B. subtilis*. Fractions obtained from *H. fulgens* showed potent activity against five Gram-negative bacteria (*Proteus mirabilis*, *P. aeruginosa*, *Enterobacter aerogenes*, *E. coli* and *Salmonella thyphi*) and two Gram-positive bacteria (*Bacillus subtilis* and *Staphylococcus aureus*).

**Table 4:** Antimicrobial activity of fractionated proteins from abalone viscera.

Hydrolyzed viscera	Fraction kDa	*P. mirabilis*	*S. sonnei*	*S. flexneri*	*P. aeruginosa*	*S. thyphimurium*	*S. thyphi*	*E. aerogenes*	*S. enteritidis*	*E. coli*	*S. aureus*	*B. subtilis*
*H. corrugata*^1^	116.25–66.2	+			+							
	21.5–6.5											+
*H. fulgens*^1^	200–31	+			+		+			+	+	+
	200–116.25									+		
	116.25–6.5	+			+			+		+		
	97.4–21.5										+	+
	66.2–31						+			+		
	45–31	+			+		+				+	+

^1^Protein concentration: 12 mg/mL for *Haliotis corrugata* and 48 mg/mL for *Haliotis fulgens*. Positive controls: Gentamycin (0.1 mg/mL), negative control (150 mM citric acid buffer, pH 5.6).

**Figure 4:**
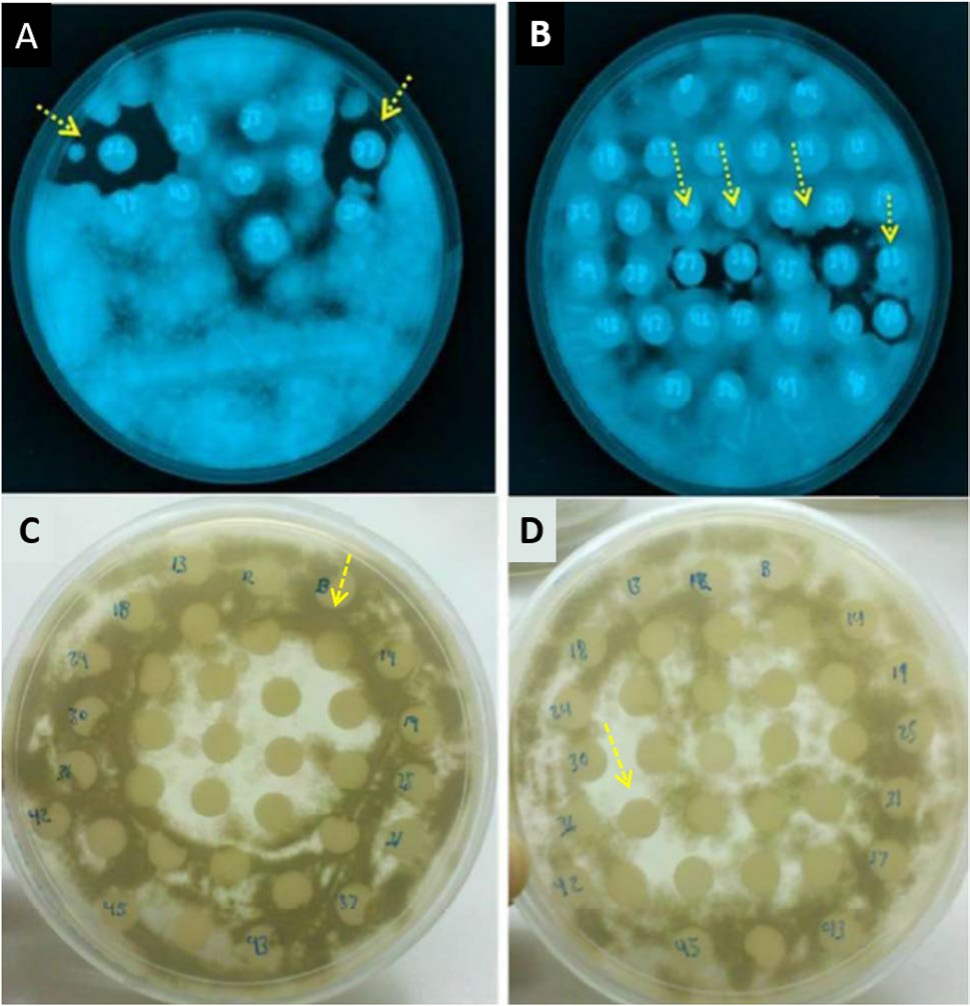
Antifungical activity of viscera fractions from *Haliotis fulgens* by agar diffusion assay. (**A**,**B**) *Aspergillus niger*, (**C**) *Alternaria alternate* and (**D**) *A. flavus*. Yellow arrows indicate inhibition growth halos. Fractions containing proteins of (**A**,**B**) 31–45 kDa, (**C**,**D**).

**Figure 5:**
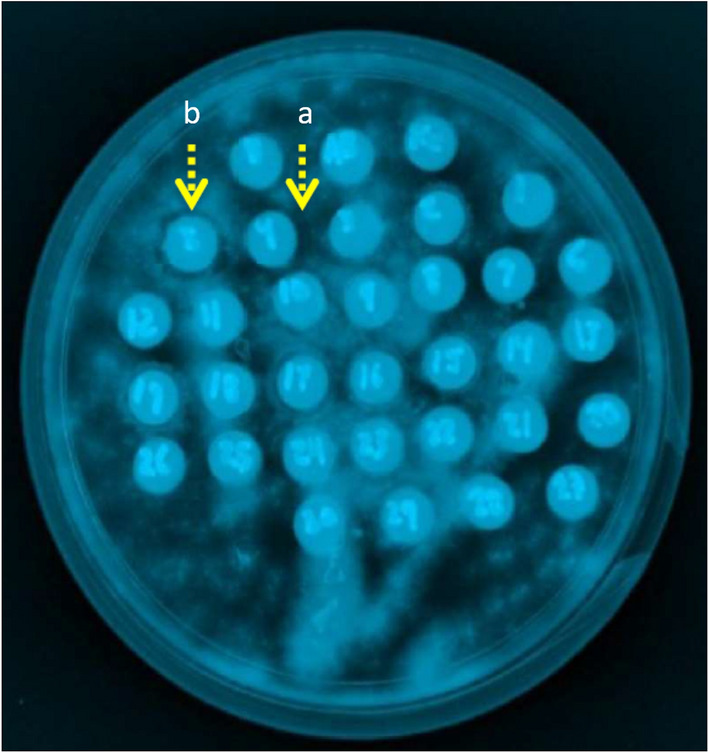
Antifungical activity of visceral fractions from *Haliotis corrugata* by agar diffusion assay against *Aspergillus niger*. Fractions with antifungical activity are marked with yellow arrows. (a) and (b) 97.4–116.25 kDa.

Antifungal activity was observed in both *H. corrugata* and *H. fulgens* protein fractions (Table 5, Figs. 4 and 5). Fractions derived from *H. corrugata,* containing protein peptides ranging from ~ 97.4–116.25 kDa, displayed inhibition specifically against *A. niger*. On the other hand, fractions from *H. fulgens* displayed inhibition against *Alternaria alternata*, *Aspergillus niger,* and *Aspergillus flavus* using fractions containing low molecular weight proteins (~ 31–66.2 kDa). None of the tested fractions for each species demonstrated antifungal activity against *Mucor* sp.

**Table 5:** Antifungal activity of fractionated proteins from viscera of *Haliotis corrugata* and *H. fulgens* abalones.

Hydrolyzed viscera	Fraction (kDa)	*Mucor sp.*	*A. alternata*	*A. niger*	*A. flavus*
*H. corrugata*^1^	116.25–66.2				
	97.4–116.25			+	
	21.5–6.5				
*H. fulgens*^1^	200–31				
	200–116.25				
	116.25–6.5				
	97.4–21.5				
	66.2–31		+	+	+
	45–31				

^1^Protein concentration: 12 mg/mL (*Haliotis corrugata*), 48 mg/mL (*Haliotis fulgens*). *Alternaria alternata, Aspergillus niger, Aspergillus flavus.*

### Cytotoxic activity

**Figure 6:**
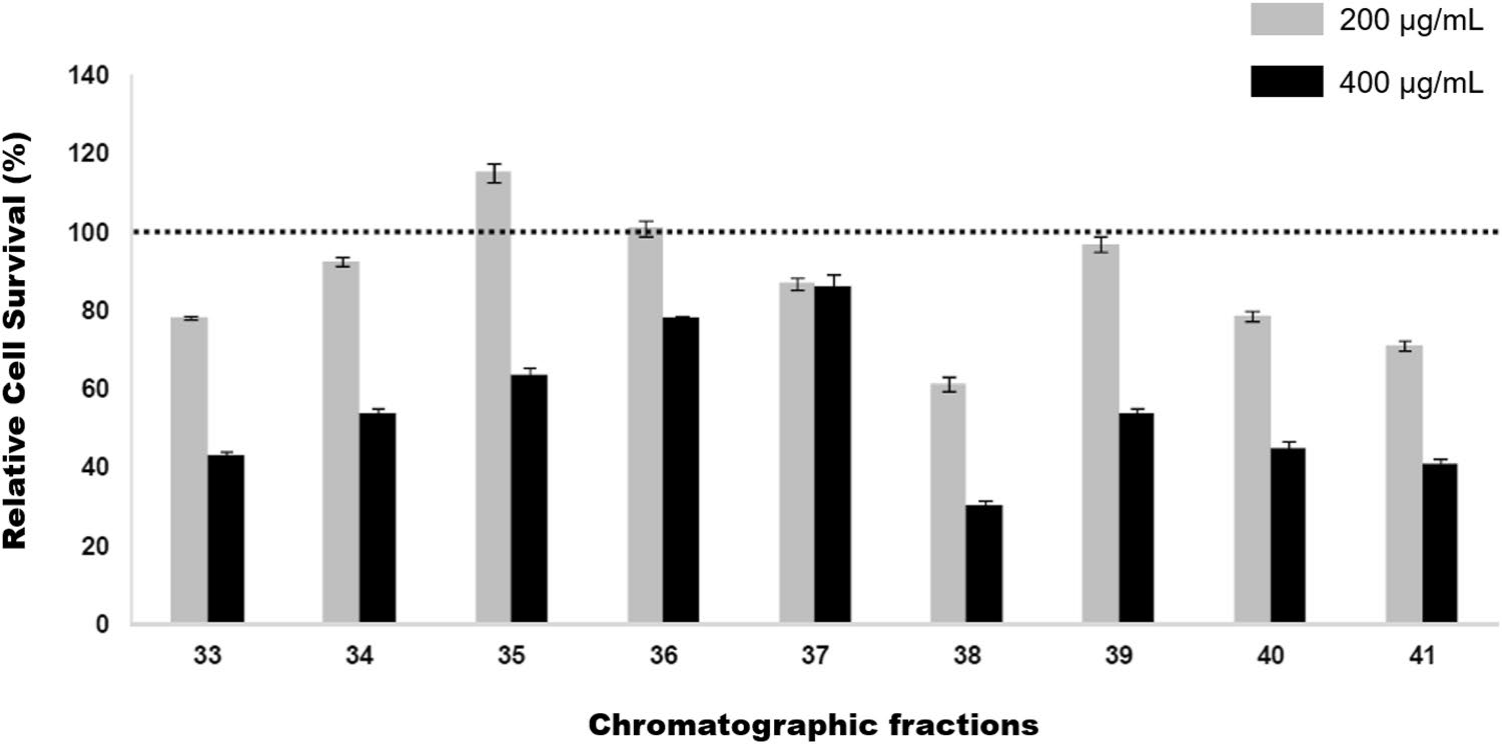
Cell viability of PC-3 cells by the MTT assay after exposure to *Haliotis fulgens* protein fractions. PC-3 cells were exposed to protein fractions containing 61.2–31 kDa proteins (200 or 400 µg/mL) for 24 h. The dotted line indicates 100% cell viability without treatment. Data are expressed as average ± standard deviation of three independent experiments.

The cytotoxic effect of visceral fractions obtained from abalones was investigated on the PC-3 cell line using the MTT assay at concentrations of 200 and 400 µg/mL as shown in Fig. 6. Fractions containing peptides of medium molecular weight (61.2–31 kDa) displayed cytotoxic activity resulting in a 50% inhibition of cell. Fractions 33, 34, and 38–41 displayed reduced cell survival ranging from 30–54% after treatment with 400 µg/mL.

### Expression of *mmp-2* and *mmp-9* in PC-3 cell lines

**Figure 7:**
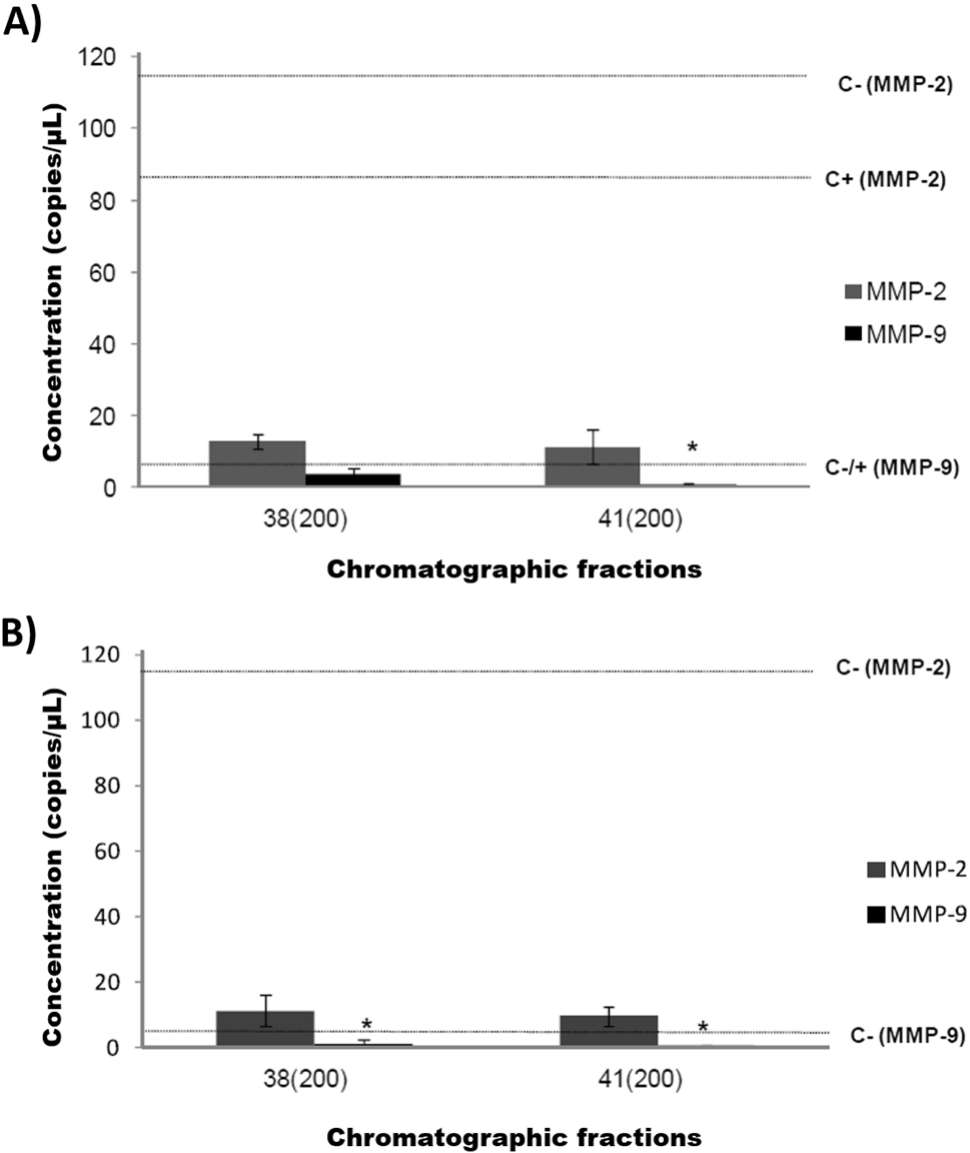
Absolute expression (copies/µL) of *mmp-2* and *mmp-9* transcripts in PC-3 cell lines after exposure to abalone fractions. PC-3 cell lines were exposed to abalone (*Haliotis fulgens*) protein fractions (61.2–31 kDa) with a protein concentration of 200 and 300 µg/mL for one hour and stimulated with (**A**) Phorbol 12-myristate 13-acetate (PMA; 10 ng/mL) for 36 h or (**B**) non-stimulated with PMA. Negative (PC-3 cell lines without treatment) and positive (PC-3 cell lines exposed to PMA) controls are indicated by dotted lines. Data are an average of three independent experiments. Asterisks indicate significant differences (p < 0.05).

The expression of *mmp-2* and *mmp-9* was tested in PC-3 cell lines, both stimulated and non-stimulated with PMA, after incubation with molecular weight proteins (66.2–31 kDa) derived from hydrolyzed visceral extracts from *H. fulgens* at concentration of 200 and 300 µg/mL (Fig. 7, Table 6). In PC-3 cell lines stimulated with PMA and treated with visceral fractions (33, 38, and 41), a low expression of *mmp-2* and *mmp-9* was observed compared to the negative and positive controls. Significant differences were observed for *mmp-9* expression with fraction 41 in contrast to both the positive and negative controls (*p* = 0.010 and *p* = 0.0005, respectively). However, no difference was observed in the *mmp-2* transcript.

**Table 6:** Absolute expression (copies/µL) of *mmp-2* and *mmp-9* transcripts in PC-3 cell lines after exposure to abalone fractions.

Sample	Stimulated with PMA		Non-stimulated with PMA	
	Concentration (copies/µL)		Concentration (copies/µL)	
	*mmp-2*	*mmp-9*	*mmp-2*	*mmp-9*
33 (300)	–	3.1	11.2	1.3*
38 (200)	12.7	3.7	–	–
41 (200)	11.3	0.9*	9.6	1.3*
Ctrl ( +)	74.2	4.3*	74.2	4.3*
Ctrl (−)	116.0	4.5*	116.0	4.5*

PC-3 cell lines were exposed to three *Haliotis fulgens* abalone protein fractions (61.2–31 kDa) with a protein concentration of 200 and 300 µg/mL for 1 h and stimulated with Phorbol 12-myristate 13-acetate (PMA; 10 ng/mL) for 36 h. Data are an average of three independent experiments.

Asterisks indicate significant differences (*p* < 0.05).

Unstimulated PMA PC-3 cell lines with fractions 33 and 41 (300 and 200 µg/mL) displayed downregulation of both gelatinases in contrast to negative controls, with significant differences for *mmp-9* transcript (*p* = 0.05). Fraction 33 with 300 µg/mL led to a 90.3% reduction of *mmp-2* and 71% for *mmp-9* (*p* = 0.007). Fraction 41 with 200 µg/mL displayed a reduction of 91.7% and 84.4% for *mmp-2* and *mmp-9*, respectively.

## Discussion

Bioactive peptides can be involved in diverse biological functions depending on their amino acid composition and molecular size. During gastrointestinal digestion, proteolytic enzymes can release bioactive peptides with potential physiological benefits^20^. In vitro gastrointestinal digestion of marine organisms produces larger quantities of bioactive peptides that are as potent as natural products^31^. Various strategies have been employed to obtain bioactive peptides in vitro, including exposure to heat, acids, proteolytic enzymes, and more recently subcritical water extracts^18^. Peptides released by enzymatic hydrolysis represent a high-cost option but provide greater precision in controlling the degree of protein hydrolysis^41^. Food-grade proteolytic preparations, such as Alcalase 2.4L, Flavourzyme 500L/1000L, Corolase PP, and Promod 144MG, have been widely used for protein hydrolysates^42–44^. However, differences in specificity among proteolytic preparations can lead to variation in the production of low molecular weight peptides^45^, which also dependent on the protein source being hydrolyzed. In this study, treatment with Wobenzym (Societe des Produits Nestle S.A. Vevey Switzerland) resulted in significant changes in the SDS-PAGE profile, reflecting extensive hydrolysis of medium molecular weight proteins (~ 31–60 kDa) from abalone viscera. However, it appears that Wobenzym does not have sufficient specificity to completely hydrolyze the high molecular weight proteins (> 66 kDa), despite containing a diverse range of cysteine and serine proteases, such as bromelain, papain, trypsin, and chymotrypsin enzymes.

Protein hydrolysates are a valuable source of peptides and amino acids with bioactive properties due to their physicochemical characteristics^46^. The bioactivity of peptides mainly depends on their structure, molecular mass, and the physicochemical characteristics of the amino acids within the sequence. The hydrolysate obtained from abalone viscera yielded peptides with high molecular weight (~ 200–116.25 kDa), medium molecular weight (~ 116.25–66.25 kDa), and low molecular weight (~ 66.2–31.0 kDa) molecular weight peptides. Medium and low molecular weight peptides have been related with antimicrobial activity in various marine species, including the oyster *Crassostrea gigas*^47^, marine snail *Cenchritis muricatus*^48^, and mussel species *Perna viridis* and *P. perna*^49^. Aqueous extracts from marine mollusks have shown inhibitory activity against Gram-positive and Gram-negative strains, such as *Klebsiella pneumoniae* and *Salmonella typhi* (*Melo melo*^50^, *Vibrio parahaemolyticus* and, *Staphylococcus aureus* and *Candida albicans* (*Babilonia spyrata*^51^), *E. coli, S. aureus*, *B. subtilis, S. pneumonia,* and *P. aeruginosa*, (*G. paradoxa*^52^). In this study, extracts of *H. fulgens* exhibited a notably wider spectrum of antibacterial inhibition compared to *H. corrugata*. Low molecular weight proteins from *H. fulgens* displayed inhibition against *P. mirabilis*, *P. aeruginosa*, S. *thyphimurium*, *S. thyphi*, *E. aerogenes*, *E. coli*, *S. aureus*, and *B. subtilis*, while peptides from *H. corrugata* in the range of 21.5–6.5 kDa were only effective against *B. subtilis*. High molecular weight peptides (116.25–66.2 kDa) from *H. corrugata* showed inhibtion solely against *P. mirabilis*, whereas high molecular weight peptides (200–116.25 kDa) from *H. fulgens* exhibited inhibition against *E. coli*. Interestingly, none of the abalone extracts demonstrated inhibitory effects on Gram-negative bacteria such as *S. sonnei*, *S. flexneri*, *S. thyphimurium,* and *S. enteritidis*.

In contrast to the antibacterial activity observed in the abalone extracts, the antifungal activity was restricted to *Alternaria alternate*, *Aspergillus nigel,* and *A. flavus* using medium molecular weight peptides (66.2–31 kDa) from *H. fulgens,* and *A. niger* using high molecular weight peptides (97.4–116.25 kDa) from *H. corrugata*. Similar antifungal activity has been reported in *M. melo* against *T. mentagarophytes* and *A. niger*^50^, *Patella rustica* against *Candida albicans*^52^, *Cenchritis muricatus* against *Botrytis cinerea*, *Fusaryum oxysporum*, *Aspergillus niger*, *Candida albicans* and, *Cryptococcus neoformans*^53^ and, *B. spirata* against *C. albicans*^51^. Despite previous investigations, the antifungal activities of tissue extract from mollusks including gastropods, are limited. The results of this study provide evidence of the potential of visceral extracts as antimicrobial agents.

Antimicrobial peptides exert their antibacterial activity through various mechanisms. The main mechanism involves the attraction, attachment, insertion, and orientation of peptides^54^. Initially, it was believed that the bactericidal mechanism of positively charged antimicrobial peptides relied solely on their interaction with negatively charged phospholipids in bacterial cell membranes. However, recent studies have revealed that antimicrobial peptides can directly eliminate pathogens by binding to targets outside the cell membrane, such as cell walls or intracellular components^55^.

A common characteristic of antimicrobial peptides' antibacterial effects is their requirement to come into contact or interact with the bacterial cell surface before exerting their action. Once bound to bacteria, the peptides primarily rely on insertion into the bacterial cell membrane to execute their bactericidal effect. This insertion disrupts the integrity of the cell membrane, leading to intracellular material leakage and disruption of the transmembrane potential. Consequently, this causes abnormal physiological processes and ultimately leads to cell death^56,57^. The low inhibition presented by abalone peptides on Gram and positive bacteria, compared to positive controls in this study suggest that abalone peptides possess low electrostatic attraction for membrane interaction or hydrophobic properties, allowing a limited insertion of the peptide into the membrane, possibly due to the production of large molecular weight proteins produced in vitro*.* Both electrostatic and hydrophobic interactions are known to be essential for antimicrobial activity^28^. However, this could be also an effect of the low concentration of the peptide responsible for antimicrobial activity in the fractions tested. Thus, further investigations are necessary to purify these active compounds and elucidate their identity, chemical composition, and mode of action as antimicrobial peptides.

Cancer is a chronic disease that results in abnormal cell growth in body tissues, leading to destruction of normal cells. The increasing worldwide cases of cancer-related deaths has prompted researchers to search for potential anticancer drugs. Extracts from marine mollusk have exhibited promising cytotoxic activity against cancer cells lines. For instance, the blue copper oxygenated respiratory protein derived from the hemolymph of the *Rapana thomasiana* demonstrated significant inhibition of cervical cancer cells (59%) and ovarian cell lines (44%), comparable to Tamoxifen, a commercially available anticancer drug^58^. Moreover, the cyclodepsipeptide Kulokekahilide-2 displayed significant cytotoxic activity against A549, K562, and MCF7 cell lines. It has been demonstrated that the cyclization of the depsipeptide contributes to its cytotoxic properties^59^.

In this study, cytotoxic activity of chromatographic fractions (33–41) from abalone was evaluated against prostate cancer (PC-3) cell line using two different concentrations. The fractions containing 200 µg/mL showed the highest cell survival rate, except for fractions 37, which exhibited 80% cell survival at both concentrations (200 and 400 µg/mL). This significant cytotoxic activity was accompanied by a reduction of matrix metalloproteinase (MMP) expression, *mmp-2* and *mmp-9*, in the PC-3 cell line, even when stimulated with PMA. MMP proteins are recognized as biomarkers for various diseases, with *mmp-9* being overexpressed in several types of tumors (carcinoma, breast, ovarian and cervical cancer, among others)^60,61^. MMPs play a crucial role in degrading the extracellular matrix (ECM), which consists of collagen, enzymes, and proteins that inhibit tumor cell metastatiss^62^. Similar results have been reported for the treatment of mice with visceral extract from the abalone *Haliotis discus hannai*, resulting in a significant inhibition of tumor metastasis through the regulation of Cox-2, EGF, VEGF, and FGF expression levels^23^. Cytotoxic activity against HT-29 cells has also been observed in the visceral extracts obtained from the same abalone species using different solvents^63^. More recently, the isolated peptide KVEPQDPSEW (AATP) has shown the ability to suppress the gene and protein expression of MMP2 and MMP9 in the HT1080 cell line by blocking MAPKs and NF-κB pathways^64,65^. These findings suggest that peptides derived from abalone, including those from *H. fulgens* and *H. corrugata*, possess significant antimicrobial, cytotoxic and anticancer activities making them potentially valuable for pharmacological purposes. However, further studies are required to isolate the responsible peptides for these activities and to elucidate their mechanisms of action, which can be tested in clinical trials.

## Conclusions

From the previous observations, antibacterial and cytotoxic activities accompanied by decrease in *mmp*-2 and *mmp*-9 gene expression suggest that peptides obtained from hydrolyzed abalone viscera possess novel bioactivity against Gram-positive and negative bacteria but limited to few fungal strains. Moreover, peptide fractions contain important regulators of *mmp*-*2* and *mmp*-*9* gene expression, reducing the cytotoxic effect on PC3 cell lines. However, further research is necessary to identify and characterize the molecule responsible for these bioactivities.

### Supplementary Information

Supplementary Figures.

Supplementary Table S1.

Supplementary Table S2.

## Data Availability

The datasets generated during and/or analyzed during the current study are available from the corresponding author on reasonable request.
