# Supplementary material for: Antimicrobial and anticarcinogenic activity of bioactive peptides derived from abalone viscera (Haliotis fulgens and Haliotis corrugata)
Source: Sci Rep. 2023 Sep 13;13:15185. doi: 10.1038/s41598-023-41491-w (PMC10499822; doi:10.1038/s41598-023-41491-w)
Supplement: Supplementary file 1 — Supplementary Figures. [file 41598_2023_41491_MOESM1_ESM.pdf]

Uncropped Figures

Fig 1 C. Uncropped figure.

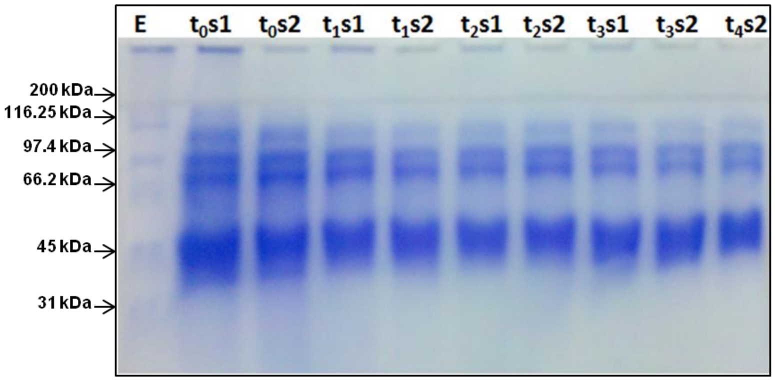

Figure 1. C) Effect of incubation time on protein recovery after hydrolysis using 2 g of Wobenzym and two centrifugation conditions. Protein recovered after 10,000 x *g* (represented by red square or S1) and 100,000 x *g* (represented by blue diamond or S2). 10% sodium dodecyl-sulfate polyacrylamide gel electrophoresis (SDS-PAGE). MM: Molecular marker. $t_{0\text{-}4}$: Time of hydrolysis of viscera from abalones. Amount of Wobenzym for hydrolysis (0.4-1.0 g).

Figure 1 D. Uncropped figure

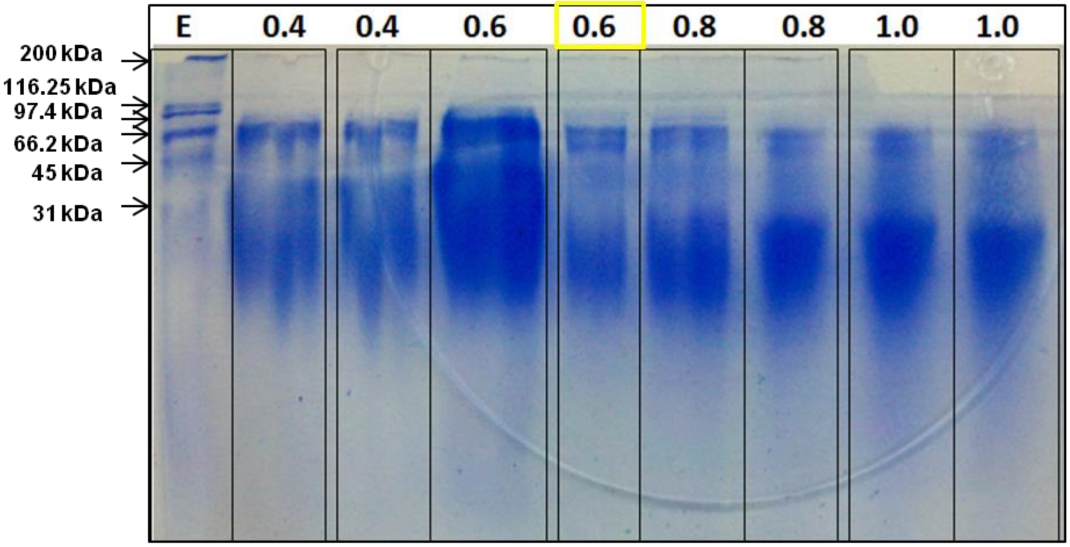

Fig. 1D) Protein recovery after hydrolysis with different amounts of Wobenzym (g). Protein recovered after 10,000 x *g* (represented by red square or S1) and 100,000 x *g* (represented by blue diamond or S2). 10% sodium dodecyl-sulfate polyacrylamide gel electrophoresis (SDS-PAGE). MM: Molecular marker. $t_{0\text{-}4}$: Time of hydrolysis of viscera from abalones. Amount of Wobenzym for hydrolysis (0.4-1.0 g).

Figure 2B. Uncropped. Triplicates are shown in the image.

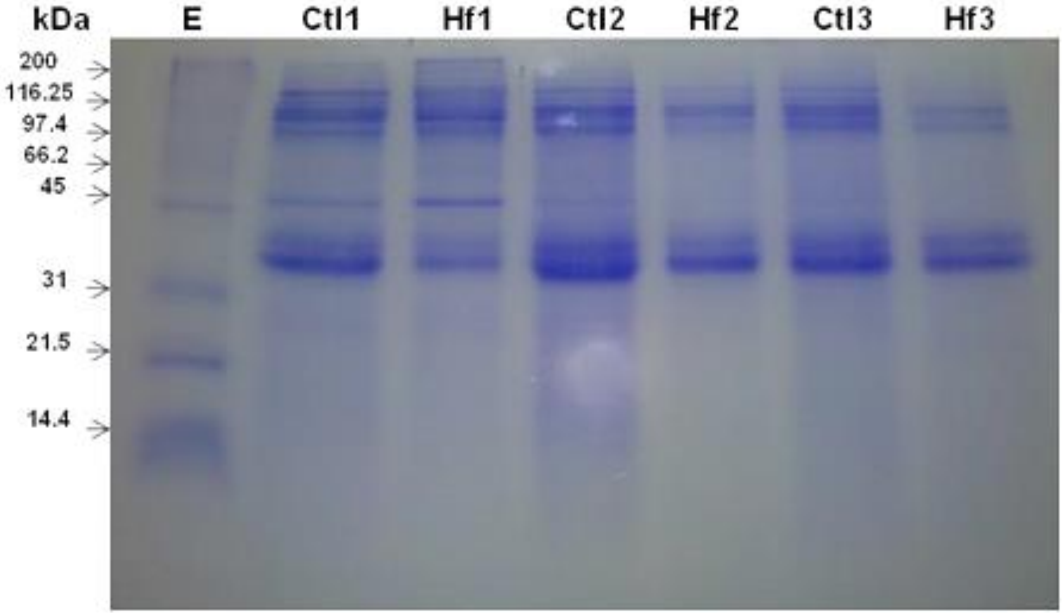

Figure 2. Gel filtration chromatographic profile of hydrolyzed *Haliotis* sp. abalone samples. **B**) 15% SDS-PAGE of unhydrolyzed (Ctrl) and hydrolyzed viscera from abalones (H). Chromatographic and SDS-PAGE are representative of three independent experiments.

Supplementary Figure 1

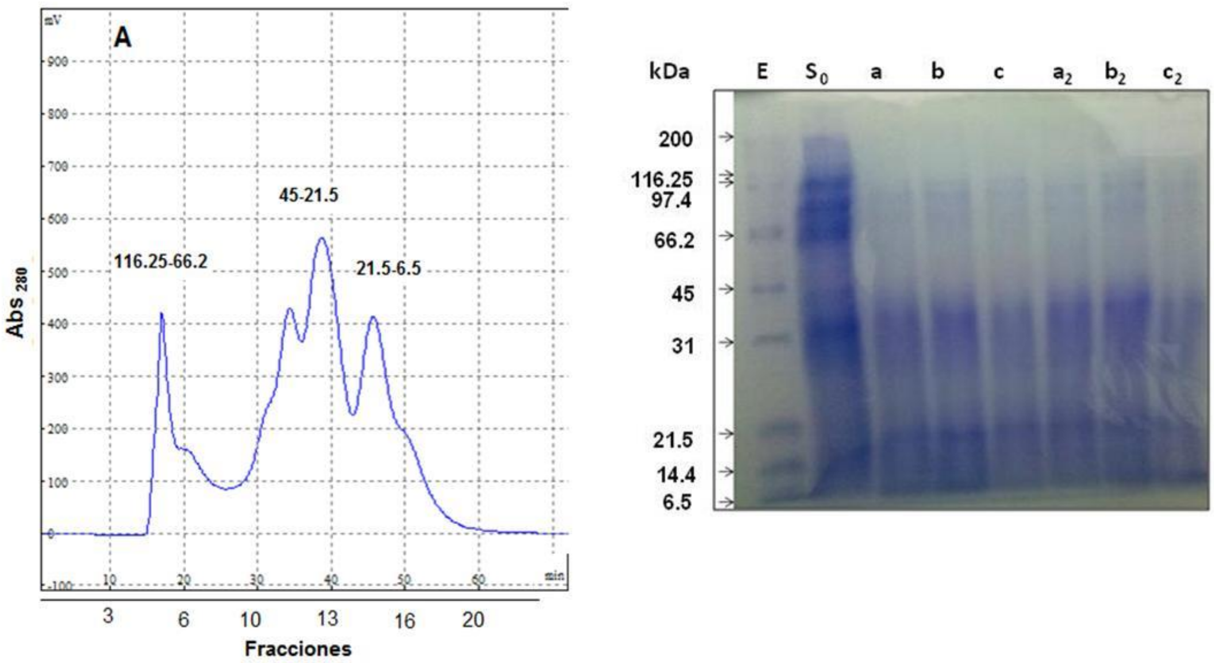

Supplementary Figure 1. Electrophoretic profile of hydrolyzed viscera of *H. fulgens* using 10 (a, a2), 12 (b, b2) and 15 (c, c2) g of Wobenzyme®. 10% SDS-PAGE of hydrolyzed viscera. E: Broad range protein marker (1610317, Bio-Rad). S0: unhydrolyzed viscera.
