# Supplementary material for: Antimicrobial and anticarcinogenic activity of bioactive peptides derived from abalone viscera (Haliotis fulgens and Haliotis corrugata)
Source: Sci Rep. 2023 Sep 13;13:15185. doi: 10.1038/s41598-023-41491-w (PMC10499822; doi:10.1038/s41598-023-41491-w)
Supplement: Supplementary file 2 — Supplementary Table S1. [file 41598_2023_41491_MOESM2_ESM.pdf]

Table S1. Wobenzym® pill content.

Enzyme	Amount per serving
Pancreatine	100 mg
Bromelaine	45 mg (112,500 UI)
Papain	60 mg (12,000 UI)
Lipase	10 mg (10,000 UI)
Amilase	10 mg (60,000 UI)
Trypsin	24 mg (60,000 UI)
Chymotrypsin	1 mg (1,000 UI)
Rutin	50 mg
Excipient cbp	One pill in 850 mg

Wobenzym® (Societe des Produits Nestle S.A. Vevey Switzerland)
