# Supplementary material for: Antimicrobial and anticarcinogenic activity of bioactive peptides derived from abalone viscera (Haliotis fulgens and Haliotis corrugata)
Source: Sci Rep. 2023 Sep 13;13:15185. doi: 10.1038/s41598-023-41491-w (PMC10499822; doi:10.1038/s41598-023-41491-w)
Supplement: Supplementary file 3 — Supplementary Table S2. [file 41598_2023_41491_MOESM3_ESM.docx]

**Supplementary Table I**. In *vitro* bacterial sensitivity test for 8 Gram-negative bacteria (1-8) and 2 Gram-positive bacteria (9 and 10). Positive control: 4 antibiotics^1^. Negative control: sterile distilled water^1^. Treatments: 30 discs with fractions^2^ obtained by HR-S100, sample: guts of *H. fulgens* digested with 10 g of enzyme for 24 hours at 37 ^o^C.

| ***H. fulgens***  **inhibition diameter (mm)** | | | | | | | | | | | | | | | | | | | | | | | | | | | | | |  |  |  |  |  |  |  |  |
| --- | --- | --- | --- | --- | --- | --- | --- | --- | --- | --- | --- | --- | --- | --- | --- | --- | --- | --- | --- | --- | --- | --- | --- | --- | --- | --- | --- | --- | --- | --- | --- | --- | --- | --- | --- | --- | --- |
| # | **Specie** | | **Control**  **(+)** | | | | **Control**  **(-)** | **Fraction** | | | | | | | | | | | | | | | | | | | | | | | | | | | | | |
|  |  |  | **K** | **G** | **P** | **E** | **H_2_O** | **1** | **2** | **3** | **4** | **5** | **6** | **7** | **8** | **9** | **10** | **11** | **12** | **13** | **14** | **15** | **16** | **17** | **18** | **19** | **20** | **21** | **22** | **23** | **24** | **25** | **26** | **27** | **28** | **29** | **30** |
| 1 | | *Proteus mirabilis* | 1 | 1.3 | 0 | 0.9 | 0 | 0 | 0 | 0 | 0 | 0 | 0 | 0 | 0 | 0 | 0 | 0 | 0 | 0 | 0 | 0 | 0 | 0 | 0 | 0 | 0 | 0 | 0 | 0 | 0 | 0 | 0 | 0 | 0 | 0 | 0 |
| 2 | | *Shigella sonnei* | 0 | 0 | 0 | 0 | 0 | C | C | C | C | C | C | C | C | C | C | C | C | C | C | C | C | C | C | C | C | C | C | C | C | C | C | C | C | C | C |
| 3 | | *Shigella flexneri* | 0 | 0 | 0 | 0 | 0 | C | C | C | C | C | C | C | C | C | C | C | C | C | C | C | C | C | C | C | C | C | C | C | C | C | C | C | C | C | C |
| 4 | | *Pseudomona aeruginosa* | 0.8 | 1.3 | 1 | 0.8 | 0 | 0 | 0 | 0 | 0 | 0 | 0 | 0 | 0 | 0 | 0 | 0 | 0 | 0 | 0 | 0 | 0 | 0 | 0 | 0 | 0 | 0 | 0 | 0 | 0 | 0 | 0 | 0 | 0 | 0 | 0 |
| 5 | | *Salmonella thyphimurium* | 0 | 1.2 | 0 | 0 | 0 | C | C | C | C | C | C | C | C | C | C | C | C | C | C | C | 0.1 | C | C | C | C | C | C | C | C | C | C | C | C | C | C |
| 6 | | *Salmonella thyphi* | 1.1 | 1.4 | 0 | 1 | 0 | C | C | C | C | C | C | C | C | C | C | C | C | C | C | C | 0.1 | C | C | C | C | C | C | C | C | C | C | C | C | C | C |
| 7 | | *Enterobacter aerogenes* | 0.8 | 1.3 | 0 | 0.8 | 0 | 0 | 0 | 0 | 0 | 0 | 0 | 0 | 0 | 0 | 0 | 0 | 0 | 0 | 0 | 0 | 0 | 0 | 0 | 0 | 0 | 0 | 0 | 0 | 0 | 0 | 0 | 0 | 0 | 0 | 0 |
| 8 | | *Escherichia coli* | 0 | 1.9 | 0 | 0.9 | 0 | 0 | 0 | 0 | 0 | 0 | 0.1 | 0 | 0 | 0 | 0 | 0 | 0 | 0 | 0 | 0 | 0 | 0 | 0 | 0 | 0 | 0 | 0 | 0 | 0 | 0 | 0 | 0 | 0 | 0 | 0 |
| 9 | | *Staphylococcus aureus* | 0 | 1 | 0 | 0 | 0 | C | C | 0 | 0 | C | C | C | C | C | C | C | C | C | C | C | C | C | C | C | C | C | C | C | C | C | C | C | C | 0 |  |
| 10 | | *Bacillus subtilis* | 1.5 | 1.6 | 0 | 1.2 | 0 | 0 | 0 | 0 | 0 | 0 | 0 | 0.1 | 0 | 0 | 0 | 0 | 0 | 0 | 0 | 0 | 0.1 | 0 | 0 | 0 | 0 | 0 | 0 | 0 | 0 | 0 | 0 | 0 | 0 | 0 | 0 |

K: Kanamycin, G: gentamicin, P: penicillin, E/P: Streptomycin/Penicillin; Hf: *H. fulgens*. Inhibition halo in millimeters (mm); C: Growth.

^1^Positive controls (1 µg on each disc): K, Kanamycin; G, gentamicin; P, penicillin; E/P: Streptomycin/Penicillin. N: negative control: citric acid-sodium citrate buffer 20 µL.

^2^ 20 µL of each fraction.

**Supplementary Table II.** *In* *vitro* bacterial sensitivity test for 8 Gram-negative bacteria (1-8) and 2 Gram-positive bacteria (9 and 10). Positive control: gentamycin^1^. Negative control: citric acid-sodium citrate^1^ buffer. Treatments: 32 discs with the fractions^2^ obtained by HR-S100 (fractions 13 to 51), sample: guts of *H. fulgens* digested with 10 g of enzyme for 24 hours at 37 ^o^C.

K: Kanamycin; C: Growth. Hf: *H. fulgens*. Inhibition halo in millimeters (mm).

^1^Positive controls (1 µg on each disc); negative control: citric acid-sodium citrate buffer 20 µL.

^2^ 20 µL of each fraction.

| ***H. fulgens***  **inhibition diameter (mm)** | | | | | | | | | | | | | | | | | | | | | | | | | | | | | | | |  |  |  |  |
| --- | --- | --- | --- | --- | --- | --- | --- | --- | --- | --- | --- | --- | --- | --- | --- | --- | --- | --- | --- | --- | --- | --- | --- | --- | --- | --- | --- | --- | --- | --- | --- | --- | --- | --- | --- |
| # | **Specie** | **C (+)** | **C (-)** | **Fraction** | | | | | | | | | | | | | | | | | | | | | | | | | | | | | | | |
|  |  |  |  | **13** | **14** | **15** | **16** | **17** | **18** | **19** | **20** | **21** | **27** | **28** | **29** | **30** | **31** | **32** | **33** | **34** | **35** | **36** | **37** | **38** | **39** | **42** | **43** | **44** | **45** | **46** | **47** | **48** | **49** | **50** | **51** |
| 1 | *Proteus mirabilis* | 0.9 | 0 | 0 | 0 | 0 | 0 | 0 | 0 | 0 | 0 | 0 | 0 | 0 | 0 | 0 | 0 | 0 | 0 | 0 | 0 | 0 | 0 | 0 | 0 | 0 | 0 | 0 | 0 | 0 | 0 | 0 | 0 | 0 | 0 |
| 2 | *Shigella sonnei* | 0.9 | 0 | C | C | C | C | C | C | C | C | C | C | C | C | C | C | C | C | C | C | C | C | C | C | C | C | C | C | C | C | C | C | C | C |
| 3 | *Shigella flexneri* | 1.2 | 0 | C | C | C | C | C | C | C | C | C | C | C | C | C | C | C | C | C | C | C | C | C | C | C | C | C | C | C | C | C | C | C | C |
| 4 | *Pseudomona aeruginosa* | 1.1 | 0 | C | C | C | C | C | C | 0 | 0 | 0.1 | 0 | 0.1 | 0 | 0.1 | 0.1 | 0.1 | 0.1 | 0.1 | 0.1 | 0.1 | 0.1 | 0.1 | 0.1 | 0.1 | 0.1 | 0.1 | C | 0.1 | 0 | 0.1 | 0.1 | 0.1 | 0.1 |
| 5 | *Salmonella thyphimurium* | 1 | 0 | C | C | 0 | 0 | C | C | C | C | C | C | C | C | C | 0 | C | C | 0 | C | 0 | 0 | C | 0 | C | C | 0 | C | C | C | 0 | 0 | C | C |
| 6 | *Salmonella thyphi* | 1.5 | 0 | C | C | C | C | C | C | C | C | C | C | 2 | C | 2 | C | C | 0 | 0 | C | 0.1 | C | C | C | C | C | 0 | C | C | C | C | 0 | C | C |
| 7 | *Enterobacter aerogenes* | .3 | 0 | 0 | C | 0 | C | 0 | C | 0 | C | C | 0 | C | C | C | C | C | C | C | 0 | 0 | 0 | C | C | C | C | C | 0 | 0 | 0 | 0 | 0 | 0 | 0 |
| 8 | *Escherichia coli* | 0.9 | 0 | C | C | C | C | C | C | C | C | C | C | C | C | C | C | C | C | 0 | 0 | 0 | C | C | C | C | C | C | 0 | 0 | 0 | 0 | 0 | 0 | 0 |
| 9 | *Staphylococcus aureus* | 1.1 | 0 | C | C | 0 | 0 | C | C | C | C | C | C | 0.1 | 0 | 0.1 | 0.1 | 0.1 | 0.1 | 0.1 | 0.1 | 0.1 | 0.1 | 0.1 | 0.1 | 0.1 | 0.1 | 0.1 | 0.1 | 0.1 | 0.1 | 0.1 | 0 | 0.1 | 0.1 |
| 10 | *Bacillus subtilis* | 1.5 | 0 | C | C | C | 0.1 | C | C | C | C | 0.1 | 0.1 | 0 | 0 | 0 | 0 | 0 | 1 | 1 | C | 0.1 | 0 | 0 | 0 | 0 | 0 | 0 | 0 | 0.1 | 0.1 | 0 | 0.1 | 0 | 0.1 |

| ***H. corrugata***  **inhibition diameter (mm)** | | | | | | | | | | | | | | | | | | | | | | | | | | | | | | |  |  |  |  |  |
| --- | --- | --- | --- | --- | --- | --- | --- | --- | --- | --- | --- | --- | --- | --- | --- | --- | --- | --- | --- | --- | --- | --- | --- | --- | --- | --- | --- | --- | --- | --- | --- | --- | --- | --- | --- |
| **Specie** | **C (+)** | | | | **C (-)** | **Fractions** | | | | | | | | | | | | | | | | | | | | | | | | | | | | | |
|  | **K** | **G** | **P** | **E** | **H_2_O** | **1** | **2** | **3** | **4** | **5** | **6** | **7** | **8** | **9** | **10** | **11** | **12** | **13** | **14** | **15** | **16** | **17** | **18** | **19** | **20** | **21** | **22** | **23** | **24** | **25** | **26** | **27** | **28** | **29** | **30** |
| *Proteus mirabilis* | 0.9 | 1 | 0 | 0 | 0 | 0 | C | P | 0 | 0 | 0 | 0 | 0 | C | 2 | 2 | 2 | 2 | C | C | 0 | C | 0 | 2 | 0 | C | 2 | 0 | 0 | 0 | 0 | 0 | 0 | 0 | 0 |
| *Shigella sonnei* | 0 | 6 | 0 | 0 | 0 | C | C | C | C | C | C | C | C | C | C | 2 | C | 2 | C | C | C | C | C | C | C | C | C | C | C | C | C | C | C | C | C |
| *Shigella flexneri* | 0 | 4 | 0 | 0 | 0 | C | C | C | C | C | C | C | C | C | C | C | C | C | 1 | C | C | C | C | C | C | C | C | C | C | C | C | C | C | C | C |
| *Pseudomona aeruginosa* | 0 | 8 | 2 | 0 | 0 | 0.1 | 0 | 0 | 0 | 0.1 | 0 | 0 | 0 | 0 | 0 | 0 | 0 | 0 | 0 | 0 | 0 | 0 | 0 | 0 | 0 | 0 | 0 | 0 | 0 | 0 | 0 | 0 | 0 | 0 | 0 |
| *Salmonella thyphimurium* | 0 | 8 | 0 | 0 | 0 | C | C | 0 | 0 | C | C | C | C | C | C | C | C | C | C | C | C | C | C | C | C | C | C | C | C | C | C | C | C | C | C |
| *Salmonella thyphi* | 10 | 10 | 2 | 0 | 0 | C | C | C | C | C | C | C | C | C | C | 2 | C | 2 | C | C | C | C | C | C | C | C | C | C | C | C | C | C | C | C | C |
| *Enterobacter aerogenes* | 0 | 8 | 0 | 0 | 0 | C | C | 0 | 0 | C | C | C | C | C | C | 2 | C | 2 | C | C | C | C | C | C | C | C | C | C | C | C | 0 | C | C | C | C |
| *Escherichia coli* | 0.9 | 1.1 | 0 | 1.9 | 0 | C | C | C | C | C | C | C | C | C | C | C | C | C | C | C | C | C | C | C | C | C | C | C | C | C | C | C | C | C | C |
| *Staphylococcus aureus* | 0 | 1.1 | 0 | 0 | 0 | C | C | 0 | 0 | C | C | C | C | C | C | C | C | C | C | C | C | C | C | C | C | C | C | C | C | C | C | C | C | 0 |  |
| *Bacillus subtilis* | 5 | 6 | 0 | 3 | 0 | C | C | C | C | C | C | C | C | C | C | 0.9 | 0.9 | 0.9 | 0.9 | 0.9 | 0.9 | 0.9 | C | 0.9 | C | C | C | C | C | C | C | C | C | C | C |

**Supplementary Table III:** *In vitro* bacterial sensitivity test for 8 Gram-negative bacteria (1-8) and 2 Gram-positive bacteria (9,10). Positive control: 4 antibiotics. Negative control: sterile distilled water. Treatments: 30 discs with the fractions obtained by HR-S100, sample: guts of *H. corrugata* digested with 10 g of enzyme for 24 hours at 37 ^o^C.

K: Kanamycin, G: gentamicin, P: penicillin, E/P: Streptomycin/Penicillin; Hc: *H.*  *corrugata*. C: Growth. Inhibition halo in millimeters (mm).

^1^Positive controls (1 µg on each disc): negative control: sterile distilled water.

^2^ 20 µL of each fraction.
